# Human U87 glioblastoma cells with stemness features display enhanced sensitivity to natural killer cell cytotoxicity through altered expression of NKG2D ligand

**DOI:** 10.1186/s12935-017-0397-7

**Published:** 2017-02-10

**Authors:** Se-Jeong Oh, Jung-In Yang, Ok Kim, Eun-Jung Ahn, Woo Dae Kang, Jae-Hyuk Lee, Kyung-Sub Moon, Kyung-Hwa Lee, Duck Cho

**Affiliations:** 1 Department of Neurosurgery, Chonnam National University Hwasun Hospital and Medical School, Hwasun, Jeollanam-do South Korea; 2 Department of Pathology, Chonnam National University Hwasun Hospital and Medical School, Hwasun, Jeollanam-do South Korea; 3 Department of Obstetrics and Gynecology, Chonnam National University Hwasun Hospital and Medical School, Hwasun, Jeollanam-do South Korea; 4 Department of Laboratory Medicine and Genetics, Samsung Medical Center, Sungkyunkwan University School of Medicine, Seoul, South Korea

**Keywords:** Cancer stem cells, Glioblastoma, Immunotherapy, Natural killer cell, U87

## Abstract

**Background:**

Glioblastoma (GBM) is one of the most lethal tumors with a poor prognosis. Its inevitable recurrence is frequently explained by the presence of cancer stem cells. We aimed to show that human GBM cells with stemness features are more sensitive to natural killer (NK) cells than GBM cells without stemness characteristics.

**Methods:**

Natural killer cell cytotoxicity was measured using flow cytometry in neurosphere-forming U87 GBM cells cultured with neurobasal media (NBE condition) and compared with that in serum-cultured U87 GBM cells (serum condition). Cytotoxicity was examined after addition of blocking NKG2D monoclonal antibodies. The expression profile of NK ligands of NK cells were investigated by reverse transcription polymerase chain reaction and western blot analysis in the U87 GBM cells in both conditions.

**Results:**

NBE U87 cells showed higher cytotoxicity to NK cells than serum U87 cells did (55 vs 35% at an effector to target cell ratio of 5:1). The increased cytotoxicity was diminished in NBE U87 cells by a larger gap than in serum U87 cells by adding NKG2D blocking antibodies. Of the NKG2D ligands, the expression of ULBP1 and ULBP3 was relatively increased in NBE U87 cells compared to serum U87 cells.

**Conclusions:**

U87 GBM cells with stemness features demonstrate increased cytotoxicity to NK cells in association with altered NKG2D ligand expression of NK cell activating receptor. Applying immune modulation to GBM treatment may be a promising adjuvant therapy in patients with intractable GBM.

## Background

Gliomas comprise a group of central nervous system (CNS) tumors with characteristics of neuroglial cells, such as astrocytes and oligodendrocytes. Glioblastoma (GBM), the most malignant type of glioma [World Health Organization (WHO) grade IV], are lethal neoplasms with a poor prognosis despite extensive treatments involving surgical resection with various combinations of radiotherapy, chemotherapy, and possible immunotherapy [1]. The existence of cancer stem cells in gliomas has been pointed out as one of culprits of the resistance of the conventional therapies that preludes their metastatic spread and recurrence [2]. Many efforts are being made to target cancer stem cells, particularly by improving immunotherapy using natural killer (NK) cell cytotoxicity against the tumors [3].

Natural killer cells can directly kill target cells via the perforin-granzyme pathway, antibody-dependent cellular toxicity, and death receptor ligand-induced apoptosis [3]. These mechanisms are tightly regulated by a balance between activating and inhibitory signals; NK cells can attack cells with decreased inhibitory signals, increased activating receptors, or both. Among them, the interaction between NK receptor group 2, membrane D (NKG2D) activating receptor and its ligand, such as MHC I-related chain (MIC) A/B and UL16 binding protein (ULBP) 1–5, seem to play a vital role in NK cell cytotoxicity [4] against cancer cells as ‘induced self’, although the tumors can secret these ligands as a soluble form to evade the immune surveillance.

Natural killer cell-mediated immunotherapy appears to be a promising approach to target various origins of cancer stem cells. GBM stem-like cells are sensitive to activated NK cells [5, 6]. NK cells activation has also been studied to enhance its efficacy by co-culture of genetically engineered K562 feeder cells in cytokine-containing medium [7, 8]. Additionally, the U87 GBM cell line has been extensively characterized immunologically [9].

The collective evidence indicates the merit of maximizing NK cell cytotoxicity against GBM with stem cell characteristics. However, the mechanism of NK cell cytotoxicity difference between GBM cells with stem cell-like features and serum-cultured GBM cells is not clear. In this study, we compared NK cell cytotoxicity against neurosphere-forming U87 cells (NBE U87) and serum-cultured U87 cells (serum U87). The mechanism that leads to the differential NK cell cytotoxicity between NBE U87 and serum U87 cells was explored in terms of NK cell degranulation and NKG2D–NKG2D ligand (NKG2DL) interaction.

## Methods

### Cell lines and cell cultures

The U87 human GBM cell line, as reported as isocitrate dehydrogenase (IDH)-wild type and O6-methylguanine-DNA methyltransferase (MGMT) promoter methylated [10, 11], was obtained from the Korean Cell Line Bank (Seoul, Korea). U87 cells were cultured in Dulbecco’s modified Eagle’s medium (DMEM; GibcoBRL, Gaithersburg, MD., USA) supplemented with 10% fetal bovine serum (FBS; GibcoBRL) at 37 °C in a humidified 95% air and 5% CO_2_ atmosphere, termed here ‘serum’ conditions. U87 cell line was also cultured with serum-free neurobasal medium (Invitrogen, Carlsbad, CA, USA) containing 50 ng/mL of both basic fibroblast growth factor (FGF; PeproTech, Rocky Hill, NJ, USA) and epidermal growth factor (EGF; PeproTech) supplemented with N2 and B27 supplement without vitamin A and l-glutamine, which are termed ‘NBE’ conditions.

### Cytokines and antibodies

Recombinant human interleukin (IL)-2, IL-15 and IL-21 (PeproTech) were used to expand NK cells. Fuorescein isothiocyanate (FITC)-conjugated anti-human CD3 and phycoerythrin (PE)-Cy5-conjugated antihuman CD56 antibodies were used to evaluate the purity of expanded NK cells. Anti-human NKG2D antibody was used to block the NK cell receptor. PE-conjugated anti-human CD107a antibody was used as a surrogate marker of degranulation. All the antibodies were from BD Biosciences (San Jose, CA, USA).

### Flow cytometry-based NK cytotoxicity assay

Flow cytometric cytotoxicity assay using calcein acetoxymethyl ester (CAM)-stained expanded NK cells were performed as previously described [12]. U87 target (T) cells were prepared in serum and NBE conditions. Propidium iodide (PI) was added to detect target cell death. The generation of ex vivo expanded NK cells was performed as described previously, with a slight modification [8, 13, 14]. Peripheral blood mononuclear cells (PBMCs) were co-cultured with 100 Gy gamma-ray irradiated K562 (ATCC), in a 24-well tissue culture plate in the presence of IL-2 and IL-15 (PeproTech) in complete RPMI (Roswell Park Memorial Institute) 1640 medium. The target cells and effector cells were co-cultured at effector-to-target (E:T) ratios of 10:1, 5:1, and 1:1 for 4 h at 37 °C in a humidified incubator containing 5% CO_2_. Cells were acquired by means of FACSCalibur (BD Biosciences) and the data were analyzed by BD CellQuest™ Pro Software (BD Biosciences). This study was approved by the institutional review board of the Chonnam National University Hwasun Hospital (CNUHH-2016-074).

### CD107a degranulation assay

Degranulation of expanded NK cells against target GBM cells was assessed as previously described [14]. Enhanced NK cells were incubated with serum U87 and NBE U87 at an effector/target ratio of 10:1 and 5:1 for 1 h in a 96-well plate in the presence of 5 µL/mL PE-conjugated anti-human CD107a. After 1 h, monensin and brefeldin A (BD Biosciences) were added and the plate was incubated for an additional 4 h. After co-culture with target GBM cells, expanded NK cells were washed and stained with FITC-conjugated anti-human CD3 antibody and PE-Cy5-conjugated antihuman CD56 antibody on ice for 15 min. The cells were washed, fixed and then analyzed with the use of a FACSCalibur instrument.

### Cytotoxicity assay after anti-NKG2D antibody treatment

Anti-human NKG2D antibody was used to block the activating receptor NKG2D on NK cells. In blocking experiments, anti-NKG2D antibodies were added to the NK cell suspension at 10 μg/mL and incubated at 37 °C for 30 min before the addition of target cells. The number of dead cells was estimated by measuring the fluorescence intensity in medium. The fluorescence intensity in medium released spontaneously from target cells and the total maximum fluorescence intensity released from all target cells by treatment with 1% Triton X-100 were also determined.

### Reverse transcription polymerase chain reaction (RT-PCR)

Reverse transcription polymerase chain reaction was performed as previously described on 23 NK ligands [15]. Briefly, total RNA from cells was extracted using Trizol reagent (Invitrogen, Carlsbad, CA, USA). An adequate amount of RNA was reverse transcribed using LeGene Express 1st Strand cDNA Synthesis System Kit (LeGene Biosciences, San Diego, CA, USA). Primer sequences used in this study are listed in Table 1 [16]. PCR amplification of cDNA was performed using gene-specific primers and h-Taq DNA Polymerase (SolGent, Daejeon, South Korea) under the following conditions: 15 min of denaturation at 95 °C, followed by 35 cycles of denaturation for 30 s at 95 °C, annealing for 40 s at 54 °C, and extension for 40 s at 72 °C, followed by a final extension for 7 min at 72 °C. PCR cycles were limited to 35. Primers for endogenous reference gene (glyceraldehyde-3-phosphate dehydrogenase, GAPDH) were used as internal controls. PCR products were analyzed by electrophoresis on agarose gels containing ethidium bromide. The intensity of the bands were quantified by the Labworks Image Acquisition (UVP, Upland, CA).

**Table 1:** NK ligand primers used for RT-PCR

NK receptor	Ligands	Forward primer	Reverse primer	Amplicon size (bp)
NKG2D	MICA	TCTACTACGATGGGGAGCTCTT	ACTGGGGCATTGTCCATTC	66
	MICB	CTGAGAAGGTGGCGACGTA	CGAAGACTGTGGGGCTCA	111
	ULBP-1	TGGGGGATTGTAAGATGTGG	GGCCAGAGAGGGTGGTTT	83
	ULBP-2	CCGCTACCAAGATCCTTCTG	TGACGGTGATGTCATAGCAAA	105
	ULBP-3	AGGAAGAAGAGGCTGGAACC	CTATGGCTTTGGGTTGAGCTA	70
	ULBP-4	GGGAGAATTGACCCAAACG	CTTGCAGAGTGGAAGGATCAC	104
NKG2A/C/E/H	HLA-E	GGAGTGGCTCCACAAATACC	GTCAGAGATGGGGTGGTGAG	94
2DL4	HLA-G	CTGGAGAACGGGAAGGAGAT	GGGTGGCCTCATAGTCAAAG	88
2B4	CD48	CCTACATCATGAGGGTGTTGAA	ATGACAGGCTTGGGTACAGG	91
NTBA	NTBA	CCACAATTAATCATTCCAAAGAGA	TGTCATTCCCGAATTCCTCT	107
LIR2	HLA-F	ATCACCCAGCGCTTCTATGA	CTGGGGAGACTGCTCTGC	138
NKR-P1A	LLT-1	GTTCAGGGCCAGTGCATATT	GACTCTGCACCTTCCTTCACA	61
DNAM-1	PVR-1	CAACGTCACCAATGCCCTA	CTGAGTGCTCACTGGGAGGT	80
	PVR-2	GATATCTGGCTCCGAGTGCT	TCCAGTGAGCTGGACCTTCT	69
	Nectin2-1	GGCAGAGGAGGACGAAGAC	GAGCTGGGAGGGCATCTC	94
	Nectin2-2	GAGGACGAGGGCAACTACAC	GGCCTCAGCTTGGTTCTTG	108
CD100	CD72	TTCTTCACATGCGGCTCA	AGCAGCTTTTCTGATGCATTATC	67
CD28	CD80	TCCTGGGCCATTACCTTAATC	CATCTTGGGGCAAAGCAG	77
LFA-1	ICAM-1	CCTTCCTCACCGTGTACTGG	AGCGTAGGGTAAGGTTCTTGC	90
LFA-2	LFA-3	CATGTATTGTGCTGTATATGAATGGT	TCTGGCTTCCCAAGTAATGG	141
CRACC	CRACC	TCTACTATGTGGGGATATACAGCTCA	TTTAGGCTTTGACAGGTGCTC	92
FasL	Fas	GTGGACCCGCTCAGTACG	TCTAGCAACAGACGTAAGAACGA	112
TRAIL	DR4	GGGTCCACAAGACCTTCAAGT	GGGTCCACAAGACCTTCAAGT	68
	DR5-1	GTGTGTCAGTGCGAAGAAGG	GACCATCCCTCTGGGACA	90
	DR5-2	GCACCAGGTGTGATTCAGG	CCCTCTGGGACACCCTGT	143
TNF	TNFR1	CTCTCCACCGTGCCTGAC	CCAGTCCAATAACCCCTGAG	85

*CRACC* CD2-like receptor activating cytotoxic cell, *DNAM*-*1* DNAX accessory molecule-1, *ICAM*-*1* intercellular adhesion molecule 1, *LFA* lymphocyte function-associated antigen, *MIC* MHC I-related chain, *NK* natural killer, *NKG2D* NK receptor group 2; membrane D, *PVR*-*1* poliovirus receptor-1, *RT*-*PCR* real-time quantitative polymerase chain reaction, *TNF* tumor necrosis factor, *TRAIL* tumor necrosis factor-related apoptosis-inducing ligand, *ULBP* UL16 binding protein

### Western blotting

Total cellular proteins were extracted from cultured cells using RIPA Buffer (Biosolution, Korea) supplemented with protease inhibitor Cocktail (Roche, Germany). Briefly, lysates were cleared by centrifugation at 12,000 rpm for 30 min at 4 °C. Supernatant containing proteins were collected for immunoblotting, extracted proteins (20–40 μg) were separated by SDS-PAGE (6–15%) gel and then electroblotted onto Polyvinylidene Fluoride (PVDF) membranes (Amersham Hybond-P, GE-Healthcare Life Science, Pittsburgh, PA, USA). Followed by transfer membranes were blocked with 5% w/v skim milk in TBST (TBS; 0.05 M Tris, 0.15 M NaCl, pH 7.6 and 0.1% Tween20) for 1 h and then probed with primary antibodies diluted in 3% BSA in TBST for overnight. Membranes were washed in TBST and then incubated with HRP-conjugated anti-mouse or anti rabbit secondary antibodies. Membranes were detected with an electrochemiluminescence (ECL) system (Millipore). The bands were visualized by Luminescent image analyzer (FUJIFILM, LAS-4000). The following antibodies were used: ULBP1 (1:500, sc-33564, Santa cruz biotechnology, Dallas, TX, USA), ULBP3 (1:300, sc-390844, Santa cruz biotechnology).

### Statistics

GraphPad Prism version 6.00 software program for Windows (GraphPad, La Jolla, CA, USA) was used to analyze the experiments, with the data presented as the mean ± the standard error of the mean (SEM). Statistical significance was defined at *P* < 0.05.

## Results

### NK cell cytotoxicity to NBE and serum U87 GBM cells

**Fig. 1:**
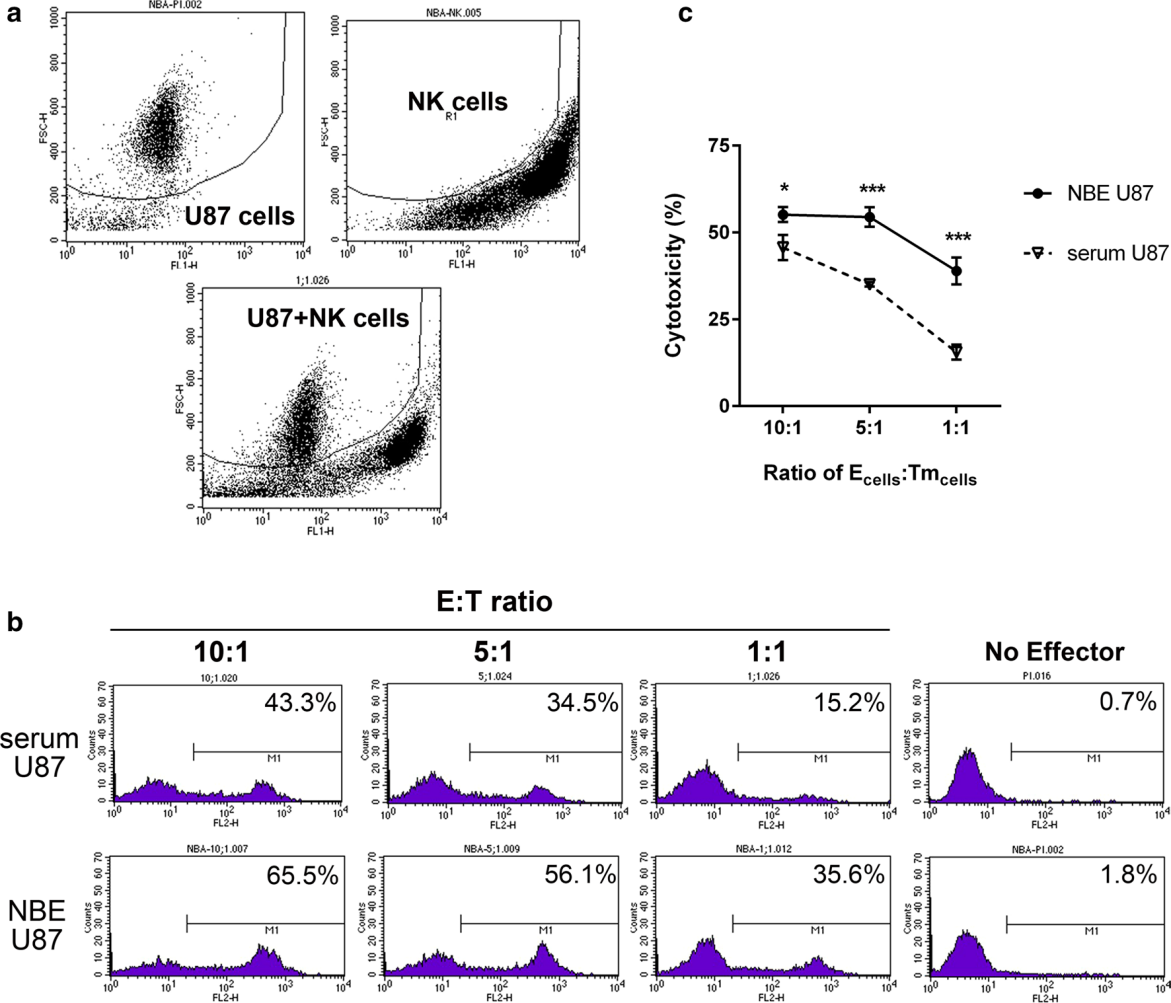
Representative flow cytometry view and cell compartment. **a** Forward scatter profiles were used to discriminate target and effector cells before (*upper panels*) and after (*lower panel*) co-culture of effector cells (NK cells) and target cells (U87 GBM cells). **b** Effector NK cells enhanced lysed target cell compartments in neurobasal medium-cultured NBE U87 cells (NBE U87) compared to serum-cultured U87 cells (serum U87) proportionally according to decreasing order of E:T ratio. **c** The average cytotoxicity levels are shown in decreasing order of E:T ratio, representing increased cytotoxicity of NK cells in NBE U87 cells compared to serum U87 cells. *Bar graphs* show the mean ± the standard error of the mean (SEM). (*P < 0.05, ***P < 0.001)

To determine whether GBM cells with stem cell characteristics (NBE U87) were more susceptible to NK cell cytotoxicity than serum U87 cells, we co-cultured effector NK cells with each corresponding NBE U87 and serum U87 cell lines (Fig. 1a). Assays were performed in triplicate under serum and NBE conditions with E:T ratios from 10:1, 5:1, to 1:1 for expanded NK cells (Fig. 1b, c). Both cell lines showed increasing cell lysis proportional to increasing amount of effector NK cells. NBE U87 cells were, however, more sensitive than serum U87 cells to enhanced NK cells, with median cytotoxicity at 5:1 effector/target ratio of 56.1 and 34.5%, respectively (*P* < 0.001).

### NK cells cytotoxicity with regard to CD107a degranulation

**Fig. 2:**
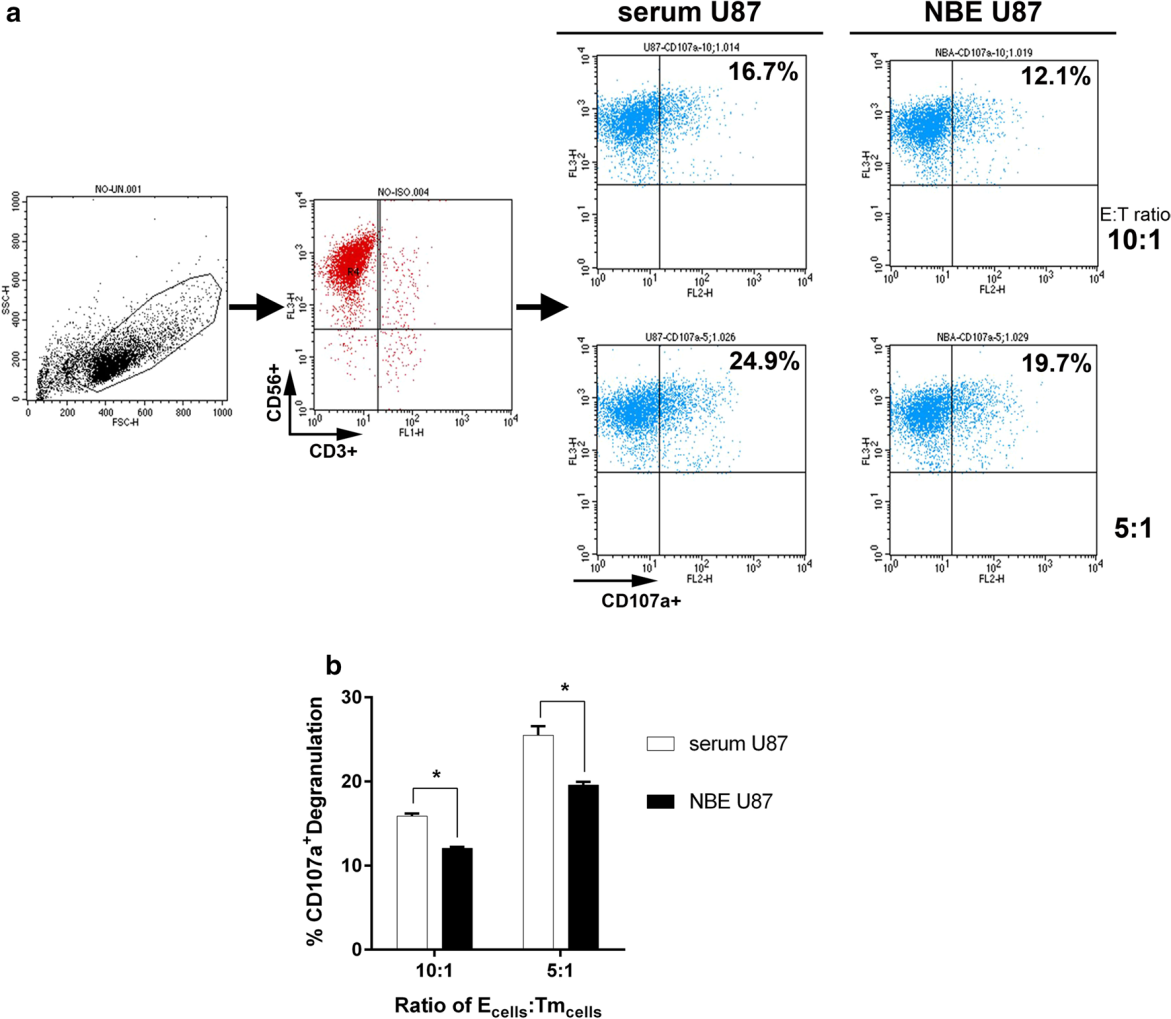
Comparison of the percentage of NK cells characterized by CD107a^+^ phenotype. **a** The purity of expanded NK cells were evaluated upon immunostaining as CD3^−^CD56^+^ cells and the percentage of CD107a^+^ NK cells were assessed by flow cytometry in both NBE U87 cells and serum U87 cells. **b** Serum U87 cells showed higher fraction of CD107a^+^ NK cells than NBE U87 cells did. *Bar graphs* show the mean ± the standard error of the mean (SEM). (*P < 0.05)

We investigated the relationship between CD107a-mediated degranulation and NK cell cytotoxicity to U87 tumor cells in each group. Using flow cytometry, we differentiated subsets of NK cells with CD56^+^CD3^−^ then those with CD107a^+^ in each co-cultured cell lines (Fig. 2a). However, the lower fraction of the CD107a^+^ NK cells was observed in NBE U87 than in serum U87, 19.7 and 24.9% respectively at a 5:1 effector/target ratio (Fig. 2b, *P* < 0.05). CD107a^+^ was not increased with a higher effector/target ratio. Contrary to the hypothetical expectation, fractions of CD107a^+^ NK cells with degranulation did not appear to explain the differential cytotoxicity between NBE U87 cells and serum U87 cells.

### NK cells cytotoxicity via NKG2D–NKG2DL interaction

**Fig. 3:**
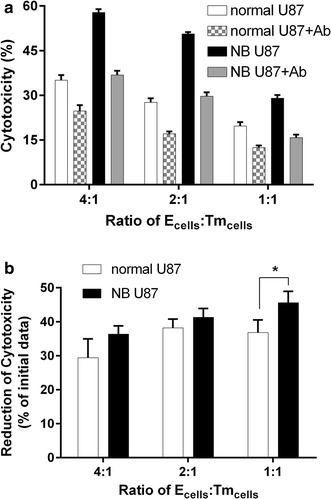
GBM cell sensitivity to NK cell lysis that occurs via NKG2D–NKG2DL interactions. **a** Cytotoxicity assays showing that GBM cells are sensitive to NK-mediated cell elimination using NKG2D blocking antibody at different E:T ratio. **b** Comparison of reduction rate between NBE U87 cells and serum U87 cells. The result showed that NK-mediated cell elimination effect using NKG2D blocking antibody is greater in NBE U87 cells, suggesting that the different cytotoxicity is mediated by NKG2D–NKG2DL interactions

Next, we measured NK cell cytotoxicity to tumor cell lines by directly neutralizing NKG2D receptor by NKG2D antibody. It was apparent that NKG2D blocking decreased NK cell cytotoxicity in both cell lines, but with a larger decrease in NBE U87 than serum U87 (Fig. 3a). The reduction of cytotoxicity was higher in NBE U87 than serum U87 with blocking NKG2D receptor with statistical significance at 1:1 of E:T ratios (Fig. 3b, *P* < 0.05).

### Expression of NK ligands by RT-PCR and western blot

**Fig. 4:**
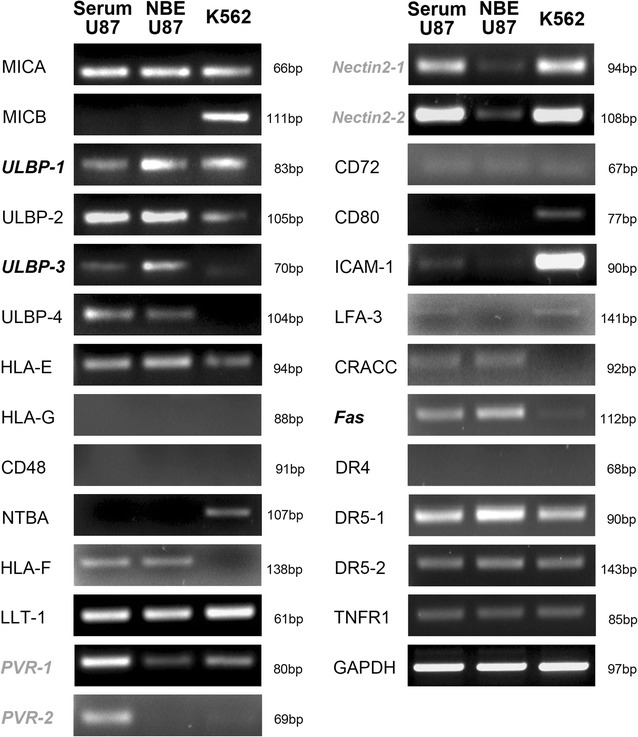
Expression of NKG2D ligands in serum U87 and NBE U87 GBM cells by RT-PCR. Increased expression levels are shown in ULBP1, ULBP3, and FAS (in *bold italic*). In comparison, the expression levels reduced in PVR-1, PVR-2, Nectin 2-1, and Nectin 2-2 (in *gray bold italic*)

**Fig. 5:**
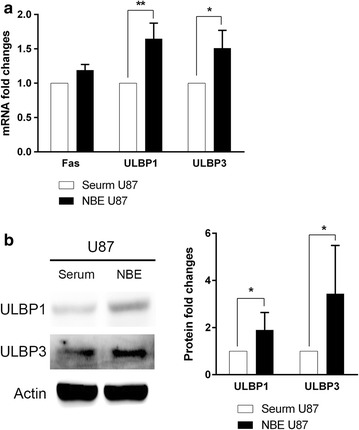
Quantification of NKG2D ligands in serum U87 and NBE U87 GBM cells. **a** ULBP1 and ULBP3 mRNA was increased in NBE U87 GBM cells. Increase of FAS mRNA was not statistical significant. **b** ULBP1 and ULBP3 protein expression was also enhanced. *Bar graphs* show the mean ± the standard error of the mean (SEM). (*P < 0.05, **P < 0.01)

Since the cytotoxicity was decreased by a larger gap in NBE U87 cells than in serum U87 cells after applying NKG2D blocking antibodies, the expression of causal NKG2D ligands and other K cell ligands on the tumor cell surfaces were explored. Serum U87 and NBE U87 cell lines exhibited different RNA profiles of NK ligands (Fig. 4). In NBE U87, the expression of NKG2D ligands including ULBP1 and ULBP3 were relatively increased (Fig. 5a, *P* < 0.01 in ULBP1 and *P* < 0.05 in ULBP3). FAS expression was also increased with marginal statistical significance. In contrast, the level of DNAX accessory molecule-1 (DNAM-1) ligands of poliovirus receptor (PVR)-1, PVR-2, Nectin 2-1, and Nectin 2-2 were decreased in NBE cells (Fig. 4). The enhanced protein levels of ULBP1 and ULBP3 were also confirmed (Fig. 5b, *P* < 0.05 in both ligands).

## Discussion

In the present study, human GBM cells with stem cell-like features (NBE U87) showed increased cytotoxicity to enhanced NK cells compared to serum-cultured GBM cells (serum U87). It was also suggested that increased cytotoxicity was mediated by NKG2D–NKG2DL interaction supported by different NK cell cytotoxicity in each groups after applying NKG2D blocking antibodies. In addition, NKG2DL expression in NBE U87 was altered in comparison of that in serum U87. Interestingly, we observed that the mechanism of different NK cell cytotoxicity with regard to stem cell-like features was not due to degranulation. As previously reported characteristics of U87 cell line, this study is focused on IDH-wild type GBM.

Activated NK cells are capable of killing different types of cancer cells including glioma cells [5–7]. Once NK cells are activated by various means including IL-2, IL-15, or PHA, they can overcome immune escape of glioma, such as HLA class I molecules, by overwhelming the activating signals [5, 6]. We used K562 cells in the presence of IL-2 and IL-15 to activate NK cells [7]. A previous study demonstrated that GBM cells with stem cell-like features were susceptible to lysis by lymphokine-activated NK cells [6], in contrast to the NK cell resistance caused by use of glioma cells cultured under non-stem cell conditions or freshly isolated NK cells [6].

In the current study, NKG2D–NKG2DL interaction played a significant role in enhanced NK cytotoxicity against glioma cell lines. Prior studies reported controversial results on the mechanistic cause of increased cytotoxicity of tumor cells with stem cell features compared to serum-cultured tumor cells. Glioma is vulnerable to NK cells via NKp44, NKp46 [5], or DNAM-1 receptors [6] and their cytotoxicity is considered minimal or to be minor via NKG2D. Proneuronal GBM cancer stem cell lines were reported to downregulate NKG2D expression on NK cells through transforming growth factor-beta-dependent suppression, providing an explanation for the reduced immune infiltration [17]. The level of NKG2DL expression in tumor cells does not appear to correlate with increasing cytotoxicity [9]. The discrepancy between the current study and previous reports can be speculated as previously pointed out [5]; the target cells used (U87 immortalized GBM cell line vs. primary cultured cell lines from GBM patients); methodological modification in generating enhanced NK cells (use of K562 feeder cells), cytokine concentration, duration of cytokine treatment); or the effector cells used (polyclonal MelanA specificT cell lines vs. enhanced NK cells after separation from PBMCs of healthy donors).

However, there have been some subtle points that suggest a mechanistic possibility similar to our results. Jung et al. [9] demonstrated the increased expression levels of NKG2D ligands in NBE U87 cells, similar to our results. Avril et al. showed mRNA analysis results that displayed expression of NKG2D ligands [5] and Di Tomaso et al. [18] also reported more commonly detectable MICA, ULBP-2, ULBP-3, and ULBP-4, although at a low levels. Also, the interaction between NKG2D and DNAM-1 on the surface of NK cells with their ligands on tumor cells was designated to be critical for NK cell cytotoxicity against sarcoma cells as well [7].

Presently, we discovered that the ligands of activating receptors of NK cells differ from NBE U87 to serum U87. Although the level of NKG2DL does not necessarily affect NKG2D mediated cytotoxicity with enhanced NK cells [9], NBE U87 showed increased cell lysis compared to serum U87. High expression of MICA and ULBP2 in both cell lines could result in basal cytotoxicity, whereas the increment of ULBP1 and 3 may contribute to relatively higher cytotoxicitiy in stem cell-like GBM.

Additionally, stem cell-like glioma may display heterogeneous NK cell ligands depending on different subtypes. NBE U87 GBM cell line used in this study showed different RNA profile compared to other studies. Castriconi et al. [6] showed increased expression of DNAM-1 ligands, PVR and nectin-2, in GBM grown in neurobasal medium whereas we observed decreased amounts of these ligands in NBE U87. In this sense, the various types of glioma could make it difficult to exploit uniformly enhanced NK cells and individualized immunotherapy is much desired.

It is interesting to note that NK cell cytotoxicity did not attribute to the degree of degranulation. Low level of CD107a^+^ fraction in degranulation assay reflects the fact that enhanced NK cell cytotoxicity does not result from perforin granzyme release. This result implies that NK cell activation with NKG2D provides different mechanisms to lyse target cells, such as inducing apoptosis or mediating other cytokines.

Enhanced NK cells provided promising results in eliminating many different tumor cell lines [7, 8]. Although NK cell cytotoxicity is lower in glioma cell lines than other types of tumors, it is still necessary to show improved cytotoxicity compared to conventional methods [5, 6]. Whether the different cytotoxicities among various tumor types are due to different NK cell ligands expression is uncertain. However, many experiments have shown that NKG2D ligands induction in different tumors including glioma cell lines give a rise in increased cell lysis [19–22], indicating that enhanced NK cell ligands in tumor cells do play role in increasing cytotoxicity.

Recent emerging evidence has supported the potential synergistic effects of therapeutic combinations against solid tumors [3]. Examples include combining NK cell therapy with radiation therapy or with monoclonal antibody therapy. Radiation-induced tissue injury increases the expression of NK activating ligands, such as NKG2D, and monoclonal antibody therapy allows NK cells to kill antibody-coated target cells through antibody-dependent cellular cytotoxicity.

## Conclusion

The results indicate that enhanced NK cell cytotoxicity against GBM stem cell populations in combination with other conventional anti-cancer therapy may be a promising treatment option in the near future. Further investigation is needed to elucidate the true nature of the enhanced sensitivity to GBM cell with stemness features by enhanced NK cells.

## Abbreviations

DNAM-1: DNAX accessory molecule-1
GAPDH: glyceraldehyde-3-phosphate dehydrogenase
GBM: glioblastoma
MIC: MHC I-related chain
NBE: neurobasal media
NK: natural killer
NKG2D: NK receptor group 2, membrane D
PBMC: peripheral blood mononuclear cell
PVR-1: poliovirus receptor-1
RT-PCR: real-time quantitative polymerase chain reaction
ULBP: UL16 binding protein
